# Therapeutic Patterns and Surgical Decision-Making in Breast Cancer: A Retrospective Regional Cohort Study in Romania

**DOI:** 10.3390/clinpract15080145

**Published:** 2025-08-05

**Authors:** Ramona Andreea Cioroianu, Michael Schenker, Virginia-Maria Rădulescu, Tradian Ciprian Berisha, George Ovidiu Cioroianu, Mihaela Popescu, Cristina Mihaela Ciofiac, Ana Maria Petrescu, Stelian Ștefăniță Mogoantă

**Affiliations:** 1 Doctoral School, University of Medicine and Pharmacy of Craiova, 200349 Craiova, Romania; 2 Department of Endocrinology, University of Medicine and Pharmacy of Craiova, 200349 Craiova, Romania; 3 Department of Oncology, University of Medicine and Pharmacy of Craiova, 200349 Craiova, Romania; 4 SF Nectarie Oncology Center, 200347 Craiova, Romania; 5 Department of Medical Informatics and Biostatistics, University of Medicine and Pharmacy of Craiova, 200349 Craiova, Romania; 6 Department of Physical Medicine and Rehabilitation, University of Medicine and Pharmacy of Craiova, 200349 Craiova, Romania; 7 Department of Radiology and Medical Imaging, University of Medicine and Pharmacy of Craiova, 200349 Craiova, Romania; 8 Department of Surgery, University of Medicine and Pharmacy of Craiova, 200349 Craiova, Romania

**Keywords:** breast cancer, surgical approach, screening

## Abstract

**Background**: Breast cancer is the most prevalent malignancy among women globally. In Romania, it is the most frequent form of cancer affecting women, with approximately 12,000 new cases diagnosed annually, and the second most common cause of cancer-related mortality, second only to lung cancer. **Methods**: This study looked at 79 breast cancer patients from Oltenia, concentrating on epidemiology, histology, diagnostic features, and treatments. Patients were chosen based on inclusion criteria such as histopathologically verified diagnosis, availability of clinical and treatment data, and follow-up information. The analyzed biological material consisted of tissue samples taken from the breast parenchyma and axillary lymph nodes. Even though not the primary subject of this paper, all patients underwent immunohistochemical (IHC) evaluation both preoperatively and postoperatively. **Results**: We found invasive ductal carcinoma to be the predominant type, while ductal carcinoma in situ (DCIS) and mixed types were rare. We performed cross-tabulations of metastasis versus nodal status and age versus therapy type; none reached significance (all *p* > 0.05), suggesting observed differences were likely due to chance. A chi-square test comparing surgical interventions (breast-conserving vs. mastectomy) in patients who did or did not receive chemotherapy showed, χ^2^ = 3.17, *p* = 0.367, indicating that chemotherapy did not significantly influence surgical choice. Importantly, adjuvant chemotherapy and radiotherapy were used at similar rates across age groups, whereas neoadjuvant hormonal (endocrine) therapy was more common in older patients (but without statistical significance). **Conclusions**: Finally, we discussed the consequences of individualized care and early detection. Romania’s shockingly low screening rate, which contributes to delayed diagnosis, emphasizes the importance of improved population medical examination and tailored treatment options. Also, the country has one of the lowest rates of mammography uptake in Europe and no systematic population screening program.

## 1. Introduction

Breast cancer is the most prevalent malignancy among women worldwide, with approximately 2.3 million new cases and 670,000 fatalities in 2022. It accounts for approximately 11–12% of all malignancies worldwide [1]. Romania’s breast cancer incidence is on the rise, with approximately 12,000 Romanian women diagnosed each year, making it the second most common cause of cancer-related mortality, following lung cancer [2]. It is important to note that Romania has always lacked a coordinated mammography screening program, resulting in a much lower detection rate than in the European Union. For example, a 2021 study found that 79% of Romanian women had never had a breast X-ray (mammogram). In consequence, Romania has a higher breast cancer mortality rate than Western Europe [3]. Breast cancer presentation is mostly determined by tumor features, not by the patient’s age. Recent studies have found that tumor size and location significantly predict nodal involvement. A study found that malignancies in the central/nipple region and a bigger primary were independent risk factors for axillary metastases [4]. The patient’s age was not an independent predictor of nodal illness in multivariable models. Lymph node positivity diminishes with advancing age [5]. International studies show that age influences surgical options. For example, mastectomy rates have historically been higher in very young patients (because of genetic risk or fear about recurrence), whereas breast-conserving surgery (BCS) is frequently feasible and safe in older people. However, age should not be the key determining factor in therapy. There is an increased emphasis on tumor biology (e.g., molecular subtype, genomic risk) and functional state over age when making therapy recommendations [6].

One of the key challenges in Romania is the absence of a national screening program for breast cancer, which contributes to delayed diagnosis and advanced disease at presentation. In this context, understanding the therapeutic patterns and the clinical decision-making process becomes essential for improving patient outcomes. This retrospective study analyzes a regional cohort of breast cancer patients from Oltenia, aiming to describe the clinical, pathological, and therapeutic characteristics of the cases. We focused on identifying patterns of care and exploring associations between age, tumor features (palpability, pain, nodal involvement), molecular subtypes, and the treatments received. Our hypothesis was that patient-related factors and tumor presentation may influence the selection of surgical approaches and the administration of adjuvant or targeted therapies, even in the absence of statistically significant group differences.

## 2. Materials and Methods

### 2.1. Study Design and Setting

This retrospective cohort study included 79 patients diagnosed with invasive breast carcinoma, treated between January 2019 and December 2024 at the Sf. Nectarie Oncology Center in Craiova, Romania. The study was approved by the Ethics Committee of the University of Medicine and Pharmacy of Craiova (Reg. no. 351/17.09.2024).

### 2.2. Eligibility Criteria and Grouping

**Figure 1:**
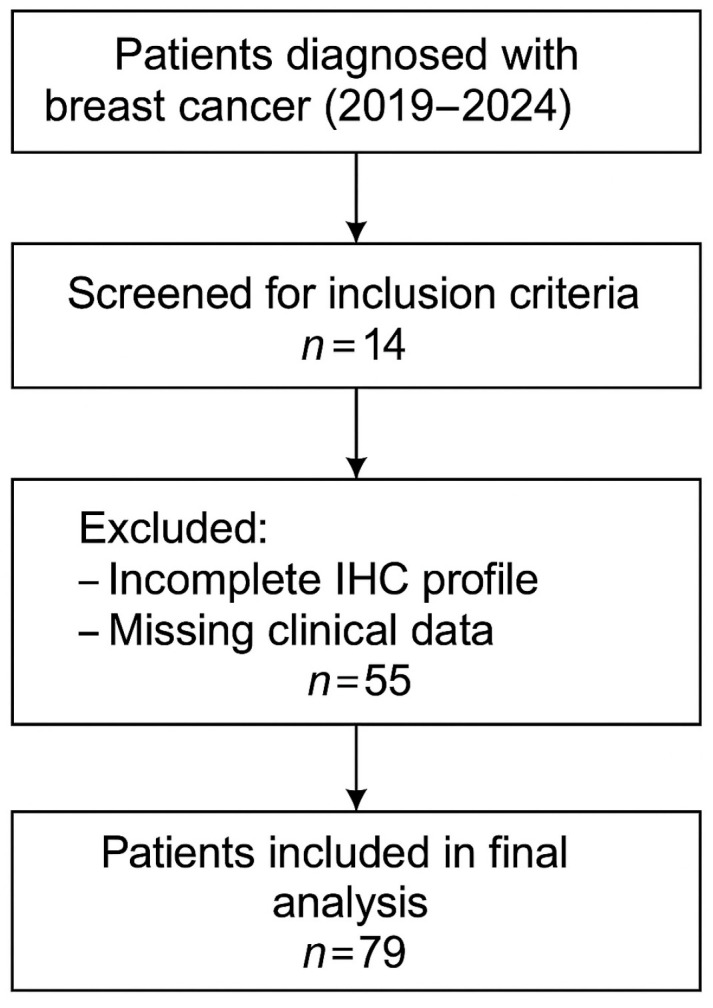
Flowchart of patient selection.

The study included patients with histologically confirmed invasive breast carcinoma who received primary surgical treatment between January 2019 and December 2024. To ensure internal consistency and enable robust biological stratification, only patients with complete immunohistochemical (IHC) profiles—both from preoperative core biopsies and postoperative surgical specimens—were considered eligible. The overall selection process is illustrated in Figure 1, which summarizes the inclusion criteria applied and the number of patients retained for final analysis.

The study group included a total of 79 patients diagnosed with breast carcinoma, who were treated at the Sf. Nectarie Oncology Center. Data were extracted on demographics (age at diagnosis), tumor histopathology (histologic subtype, grade, receptor status), clinical staging (TNM, nodal status, distant metastasis), and treatment type (surgery type, chemotherapy, radiotherapy, hormonal therapy, targeted therapy). The biological material analyzed comprised tissue fragments from the breast parenchyma and axillary lymph nodes. Some patients underwent surgery at the Emergency County Hospital of Craiova, while the rest were treated surgically in other medical centers. In addition, in certain cases, the diagnosis was made prior to surgery using a breast puncture biopsy, which is utilized for histological confirmation and to guide oncological treatment.

In patients undergoing neoadjuvant chemotherapy (NAC) for breast cancer, sentinel lymph node biopsy (SLNB) can be performed either before or after NAC. SLNB after NAC is a feasible and accurate method for axillary staging in patients with clinically node-negative disease at diagnosis, and it can help downstage microscopic nodal disease. The timing of SLNB relative to chemotherapy can affect the identification rate and false negative rate, with some studies reporting lower identification rates and higher false negative rates after NAC. However, SLNB after NAC can potentially reduce the need for axillary lymph node dissection (ALND). Some patients who were candidates for sentinel lymph node biopsy underwent lymphoscintigraphy using Technetium Tc-99m sulfur colloid. Additionally, blue dyes like isosulfan blue or methylene blue are often used in conjunction with the radiotracer for visual identification of the sentinel nodes during surgery.

**Figure 2:**
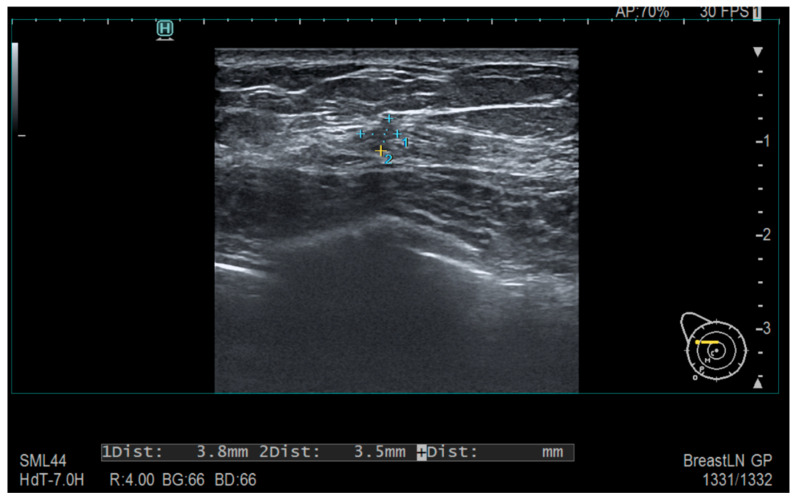
Ultrasonography—Diffusely contoured, hypoechoic area located in the external quadrant, measuring 3.8 mm × 3.5 mm.

**Figure 3:**
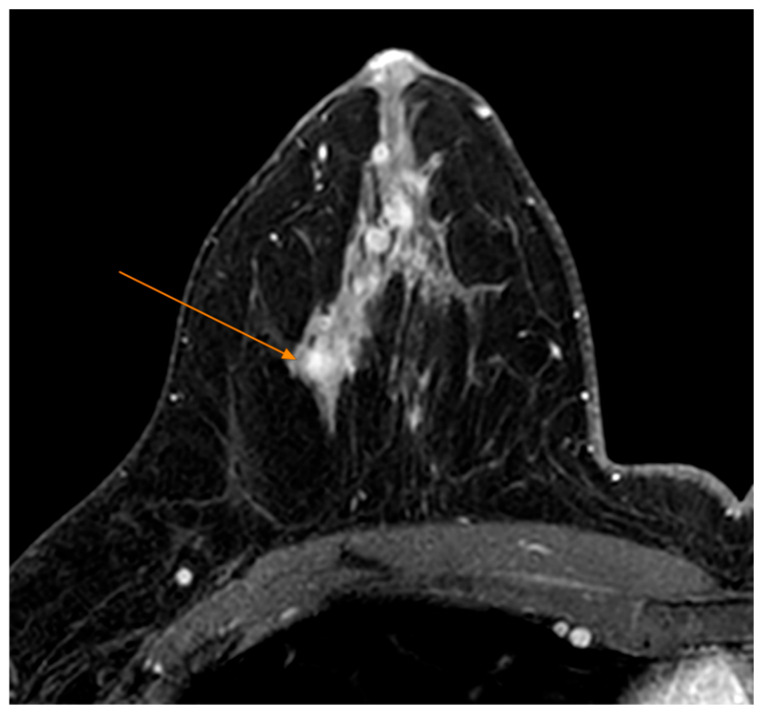
MRI Examination—T1 FS Sequence Post IV Contrast—Nodular mass-like contrast enhancement, located deep in the right breast gland, external quadrant (arrow).

**Figure 4:**
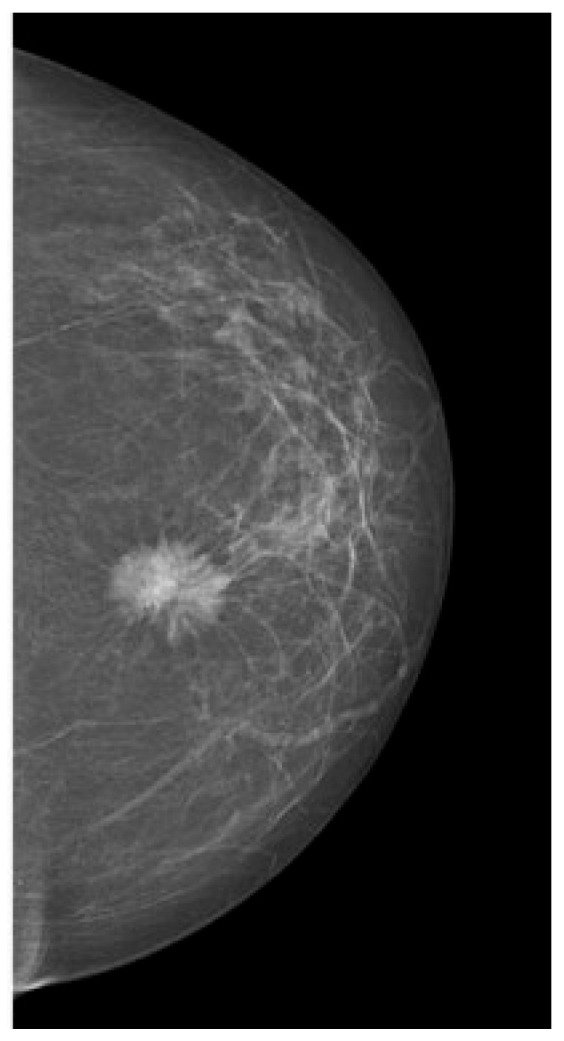
Mammography—suspicious spiculated opacities.

Tumors were classified using the WHO histologic criteria (such as invasive ductal carcinoma, invasive lobular carcinoma, ductal carcinoma in situ, and mixed histology). Age categories were used for analysis. Advanced imaging methods such as breast ultrasound (Figure 2), MRI (Figure 3), mammography (Figure 4), or CT were performed in only a subset of patients, highlighting significant shortcomings in early detection and screening practices within the healthcare system.

Statistical analysis was performed using IBM SPSS Statistics, version 26 (SPSS Inc., Chicago, IL, USA). Continuous variables were summarized using means and standard deviations, while categorical and ordinal variables were described using frequencies and percentages.

Normality of distribution was assessed using the Kolmogorov–Smirnov and Shapiro–Wilk tests. Depending on the distribution and measurement scale of each variable, appropriate statistical tests were selected. The independent samples t-test was used to compare means between two groups when normality was met, while the Kruskal–Wallis test was applied for non-normally distributed continuous variables across more than two groups.

Associations between categorical variables were tested using the Chi-square (χ^2^) test, with degrees of freedom reported where applicable. Spearman’s rank correlation coefficient was used to assess monotonic associations between ordinal or non-normally distributed continuous variables.

Logistic regression analysis was conducted to evaluate the association between chemotherapy type and the probability of undergoing mastectomy, with results reported as odds ratios (OR) and 95% confidence intervals (CI).

A two-tailed *p*-value of < 0.05 was considered statistically significant.

## 3. Results

### 3.1. Patient and Tumor Characteristics

The mean age of participants was 57.1 years, with a balanced distribution and moderate variability (SD = 11.5; IQR = 18), as presented in Table 1. The Shapiro–Wilk test confirmed normality (*p* = 0.499), allowing for parametric analysis. The study group included 78 women and only 1 man.

The most frequent histological type was invasive ductal carcinoma (78.4%), especially in patients under 50 and those aged 61–70. Invasive lobular carcinoma accounted for 17.8% of cases, most commonly in the 61–70 age group. Patients over 71 represented the smallest group (10.1%), with all tumor types being less frequent in this age category, as shown in Table 1. Statistical analysis showed no significant association between variables (χ^2^(3) = 2.86, *p* = 0.416), and the Spearman correlation was weak and nonsignificant (r = 0.016; *p* = 0.889), indicating independence and the absence of a consistent relationship.

Non-palpable tumors were observed in 30.38% of patients (24/79), while palpable tumors were observed in 69.62% of patients (55/79). The proportion of palpable tumors increased marginally when stratified by age: 64% in patients under the age of 50, 72.2% in those between the ages of 51 and 60, 71.4% in those between the ages of 61 and 70, and 75% in those over the age of 71 (Table 2). Most patients did not report pain, with the highest proportion of pain-free cases in the 61–70 age group (24.1%). Pain was more commonly reported by younger patients (<50: 16.5%), as shown in Table 2, yet the association was not statistically significant (Chi-square *p* = 0.340; Spearman r = −0.07, *p* = 0.538). Thus, age does not appear to influence pain perception or presentation significantly.

Despite the fact that age exhibited a weak or no correlation with pain, palpable tumors, or adenopathy, statistically significant interdependencies were discovered among these symptoms. Patients with palpable tumors were more likely to experience pain and axillary lymphadenopathy, and adenopathy was associated with a higher likelihood of pain (Table 3). These results emphasize that the extent and burden of the tumor are more significant than age in the context of clinical symptomatology.

The cohort contained a small number of patients who had pre-existing thyroid disease. Nevertheless, the literature consistently indicates that thyroid dysfunction is a common consequence of oncologic treatment. The risk of hypothyroidism is increased by the use of HER2-targeted or immunotherapy agents, particularly when nodal fields are targeted by radiotherapy.

**Figure 5:**
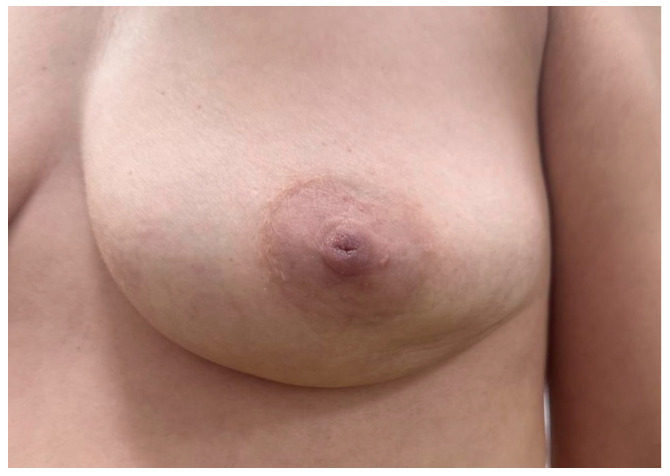
Young female presenting with a firm, well-circumscribed subareolar breast mass without visible skin changes or erythema; nipple appears centrally located with no signs of retraction or discharge.

**Figure 6:**
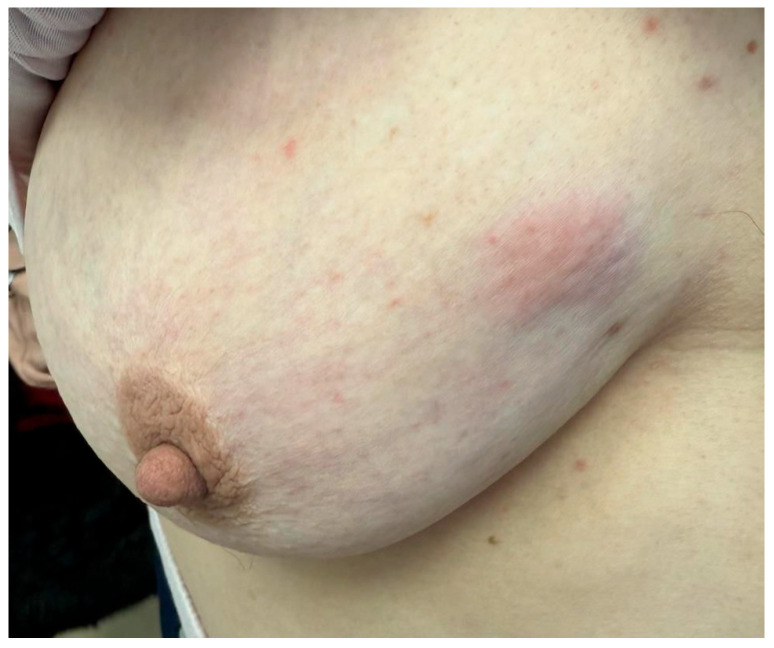
Localized area of discoloration with a violaceus hue on the upper outer quadrant of the breast, without obvious skin ulceration or nipple changes.

**Figure 7:**
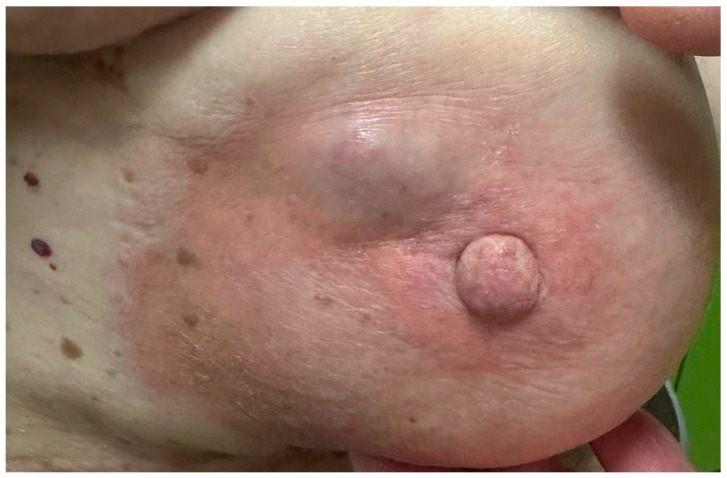
Palpable, well-defined subareolar breast mass with overlying skin erythema and slight swelling.

Examples of palpable tumors are shown in Figure 5, Figure 6 and Figure 7. The Kruskal–Wallis analysis did not reveal any significant differences between the age categories (*p* = 0.902). The feeble Spearman’s correlation between age and tumor palpability (r = 0.05, *p* = 0.634) implies that factors such as tumor size or location may be more influential than age.

### 3.2. Surgical Approach and Lymph Node Involvement

As represented in Table 4, the most common procedure identified in this study was lymphadenectomy, with 68 cases, representing 42.77% of all procedures. It is found in 86.08% of patients, suggesting an extensive use of the technique in managing oncologic pathologies. The next most frequent procedure is mastectomy, with 60 cases (37.74% of the total) being performed in 75.95% of patients. These data indicate that mastectomy remains one of the predominant therapeutic options in the treatment of severe oncologic breast cases.

In contrast, lumpectomy and sentinel lymph node biopsy are used in a significantly lower proportion. Lumpectomy was performed in 19 cases, accounting for 11.95% of all interventions and was used in 24.05% of patients. This suggests that the procedure is reserved for cases that allow conservative intervention. On the other hand, sentinel lymph node biopsy, with only 12 cases (7.55% of the total), occurs in 15.19% of patients, indicating a selective application of the method, probably for diagnostic purposes or to assess the extent of the disease. Percentages may exceed 100% because some patients underwent multiple procedures.

In terms of age distribution, younger patients (age groups under 50 and 51–60 years, respectively) were more frequently subjected to less invasive procedures such as lumpectomy and sentinel node biopsy. For example, in the under-50 age group, 9 lumpectomies, 3 sentinel node biopsies, 16 mastectomies, and 22 lymphadenectomies were performed. Although the number of mastectomies is high, lymphadenectomy is the most frequent intervention in this group. While breast-conserving procedures (lumpectomy, sentinel biopsy) remain common in younger groups (51–60), older patients (61–70 and >70) more frequently undergo mastectomy and lymphadenectomy—likely reflecting an advanced disease stage or comorbidity-driven treatment adjustments.

### 3.3. Relationship Between Chemotherapy and Surgical Decisions

A multiple logistic regression model, including age and chemotherapy (neoadjuvant and adjuvant), was applied to determine the factors influencing the likelihood of a mastectomy. The results indicate that age is the most important predictor (coefficient 0.84, Odds Ratio 2.31), followed by neoadjuvant chemotherapy (coefficient 0.26, Odds Ratio 1.3). In contrast, adjuvant chemotherapy shows a negative coefficient (−0.24, Odds Ratio 0.79), suggesting that patients who received this treatment are more likely to undergo more conservative surgery.

The distribution of surgeries was compared between patients who received chemotherapy and those who did not. Chi-square test results indicated a χ^2^ value of 3.17 and a *p*-value of 0.367, suggesting that the differences observed between the two groups were not statistically significant. Thus, chemotherapy does not appear to decisively influence the type of surgery.

A binary logistic regression model was applied to assess whether age and chemotherapy type (neoadjuvant or adjuvant) influenced the likelihood of undergoing mastectomy. The results indicated that neoadjuvant chemotherapy was associated with an increased probability of a mastectomy (β = 0.80, OR = 2.22, 95% CI: 0.44–7.63, *p* = 0.205), while adjuvant chemotherapy appeared to be associated with a reduced likelihood of a mastectomy (β = –0.79, OR = 0.46, 95% CI: 0.48–2.05, *p* = 0.224). Patient age showed a borderline statistically significant association with mastectomy (β = 0.048, OR = 1.05, 95% CI: 1.00–1.10, *p* = 0.058), suggesting a trend toward more radical surgery in older patients. Although none of the predictors reached conventional significance thresholds, the observed trends support the hypothesis that age and treatment strategies are interrelated for surgical decision-making. The overall model accuracy was 81.25%, with an acceptable goodness-of-fit.

### 3.4. Distribution of Therapeutic Strategies

In this context, the study analyzes the distribution of therapeutic strategies according to age groups and assesses the existence of statistically significant differences. Treatments investigated include adjuvant chemotherapy, adjuvant radiotherapy, neoadjuvant and adjuvant hormonal therapy, CDK4/6 inhibitors, and molecular targeted therapies. For rigorous interpretation, Chi-square tests were applied to identify associations between age and treatments, ANOVA was used to analyze differences in distribution, and logistic regression was used to determine the ability of age in predicting the chosen therapeutic strategy. Specifically, one-way ANOVA was applied to compare the distribution of patients’ age across treatment groups, revealing a statistically significant difference (*p* = 0.047).

The distribution of cases according to therapeutic strategy by age group can be seen in Table 5.

The distribution of cases according to age groups shows that most patients who did not receive adjuvant chemotherapy were under 50 years (25.3%) and 61–70 years (25.3%), followed by 51–60 years (16.5%) and over 71 years (6.3%).

The distribution of patients according to the administration of adjuvant radiotherapy does not differ significantly between age groups. Patients who did not receive this treatment are more numerous in the 61–70 age group (19.0%), followed by the under 50 age group (11.4%), the 51–60 age group (7.6%), and the over 71 age group (5.1%).

The administration of adjuvant hormone therapy is evenly distributed among age groups, with the highest values for the 61–70 age group (29.1%), followed by under 50 (27.8%), 51–60 (20.3%), and over 71 (7.6%).

The lack of notable variations between age groups suggests that this treatment is applied according to tumor biology criteria, and the Chi-square test showed no significant association (*p* = 0.169).

CDK4/6 inhibitors were administered in a small number of cases, with the highest proportions in the 61–70 (6.3%), under 50 (6.3%) and 51–60 (5.1%) age groups, while only 1.3% of patients over 71 years of age received this therapy.

A similar distribution was also observed for molecular targeted therapy, where most administrations were in the 61–70 years (6.3%) and under 50 years (3.8%) age groups, with a lower frequency among elderly patients.

### 3.5. Molecular Subtype Distribution

Among the 79 patients included in the study, molecular subtype data were available postoperatively in 44 cases. The most frequent subtype was Luminal A, identified in 24 patients (30.4%), followed by Luminal B (15 patients, 19.0%) and triple-negative breast cancer (12 patients, 15.2%). The remaining 35 cases (44.3%) lacked complete molecular classification and were grouped as “Unclassified”.

A strong association was observed between molecular subtype and the administration of adjuvant endocrine therapy. Among patients classified as Luminal A or Luminal B, hormone therapy was administered in 95.8% and 100.0% of cases, respectively. In contrast, only 20.0% of patients with triple-negative tumors received hormone therapy, consistent with their lack of hormone receptor expression. Notably, 80.0% of patients in the unclassified group also received endocrine therapy, suggesting that treatment decisions may have been guided by partial immunohistochemical data or clinical judgment.

**Figure 8:**
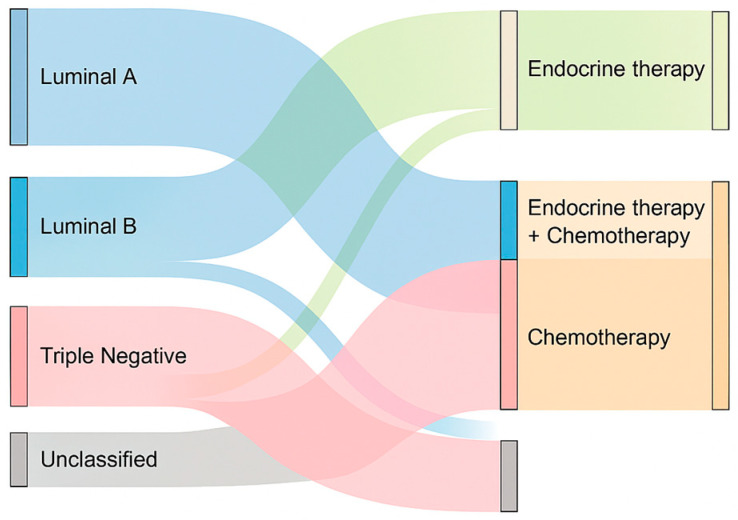
Sankey diagram illustrating the relationship between molecular subtype and adjuvant therapies.

A differential pattern was also observed in the use of adjuvant chemotherapy across molecular subtypes. Among triple-negative patients, 60.0% received chemotherapy, in line with the aggressive biology and lack of targeted options for this subtype. In contrast, only 20.8% of Luminal A patients and 40.0% of Luminal B patients underwent adjuvant chemotherapy, reflecting a more selective approach based on tumor biology and risk stratification. These findings align with current therapeutic principles, where hormone receptor–positive subtypes may receive endocrine therapy alone depending on genomic risk profiles. The unclassified group received chemotherapy in 20.0% of cases, possibly based on clinical or histological risk criteria. The relationship between molecular subtype and the type of adjuvant therapy administered is illustrated in Figure 8, which summarizes the distribution of hormone therapy, chemotherapy, and combined regimens among classified and unclassified cases.

Hormone therapy was predominantly administered in Luminal A and B subtypes, while chemotherapy was more frequent among triple-negative and unclassified cases. Combined therapy was selectively used, especially in Luminal B and unclassified tumors.

## 4. Discussion

In our retrospective cohort from Oltenia, Romania, breast cancer patients were on average in their late 50 s (mean 57.1 years), comparable to European cohorts. The male-to-female ratio (1:78) concurs with the global male BC incidence of ~1% [7].

In terms of surgical therapy, mastectomy was implemented in 75.9% instances, which is comparable to the 77.9% mastectomy rate reported by Botezatu et al. in Bucharest [7].

The relatively low incidence of breast-conserving surgery is likely a result of the advanced size/stage of the tumor at the time of diagnosis, which is partially due to the absence of screening. The literature suggests that long-term survival can be equivalent to or superior to that of a mastectomy when breast malignancies are detected early and treated with conservation plus radiotherapy. According to a recent meta-analysis, breast-conserving therapy was linked to a higher overall survival rate than mastectomy. In our situation, the potential for improved outcomes and increased eligibility for breast conservation could be achieved through earlier detection by utilizing proper screening methods [8].

We found no association between breast cancer and endocrine comorbidities at diagnosis. Interestingly, Chen et al. reported that hyperthyroidism and thyroid cancer were associated with increased breast cancer risk, while hypothyroidism was linked to lower risk [9].

The absence of pre-existing thyroid disease in our sample may be indicative of population specifics or a small sample size. It is crucial to note that hypothyroidism has been associated with the treatment of breast cancer. Falstie-Jensen et al. discovered that breast cancer survivors demonstrated a significantly higher prevalence of hypothyroidism than matched controls, notably following lymph node irradiation [10].

Our cohort’s stage distribution (many locally advanced) differs from higher-resource situations. This likely reflects the lack of routine screening: in Romania only ~9% of eligible women had any breast exam by 2020. In contrast, countries with organized mammography programs achieve much higher early-stage detection. The absence of screening likely contributed to larger tumors and the predominance of mastectomy in our series [11].

In our cohort, the distribution of surgical procedures by age revealed a clear shift toward more radical interventions with increasing age. In contrast to the above international data, our study observed the opposite trend. Younger patients (<50 and 51–60 years) were more frequently treated with breast-conserving procedures such lumpectomy and sentinel lymph node biopsy, while older patients (61–70 and >70 years) underwent a higher proportion of mastectomies and lymphadenectomies. This trend likely reflects more advanced disease stages in older age groups, or treatment individualization based on comorbidities. This interpretation was further supported by the logistic regression analysis, which showed a borderline association between increasing age and the likelihood of a mastectomy (*p* = 0.058), suggesting a possible age-related influence on surgical decision-making. This is contrary to the expectation from the literature—which suggests that fit elderly patients with early-stage, hormone-sensitive breast cancer are typically managed with less extensive surgery and breast-conserving approaches, highlighting a potential disparity in surgical management between our patient cohort and global patterns [12].

Comparatively, the international literature supports the increasing use of conservative surgery in elderly patients, particularly when tumors are early-stage and hormone receptor-positive. For instance, Natale et al. (2025) reported that over 85% of women aged ≥ 80 underwent breast-conserving surgery, with mastectomy reserved for large or multifocal tumors [6]. Interestingly, our findings differ from the ideal surgical paradigm but are consistent with real-world data from other population-based research that show similar differences. A significant example is a national research in the United Kingdom that included almost 100,000 women with early-stage breast cancer. According to Miller et al. (2024), individuals over the age of 70 are more likely to undergo mastectomy, even if they are technically qualified for breast-conserving surgery. This implies that chronological age remains a crucial component in treatment planning, often outweighing tumor biology or patient preferences [13].

This study has several limitations that must be acknowledged. First, the retrospective design and relatively small cohort size (*n* = 79) limit the statistical power to detect subtle associations, particularly in subgroup analyses. Many comparisons did not reach significance, which may reflect limited sample size rather than a true absence of effect. Second, the study was conducted at a single oncology center, potentially affecting the generalizability of results. Third, molecular subtype data were missing in a substantial proportion of patients, which may have introduced a classification bias or limited our ability to explore biologically driven treatment patterns. Finally, treatment protocols may have evolved during the 2019–2024 study period, especially regarding the availability of targeted agents, which could influence the observed therapeutic strategies.

## 5. Conclusions

Our analysis of 79 breast cancer patients from Oltenia reveals several key patterns. The predominance of invasive ductal carcinoma (approximately three-quarters of cases) aligns with global and regional data: invasive ductal carcinoma is routinely reported as the most common subtype.

It is crucial to note that we did not identify any statistically significant correlations between the primary clinical variables. Nodal status was not substantially associated with metastasis at diagnosis (possibly due to the fact that some patients with nodal disease had early detection, while a few without nodes had already experienced a hematogenous spread). This lack of association suggests that stage advancement is partly random in our population. Statistical studies revealed that patient age had no significant influence on therapy selection, despite the fact that clinical practice often modulates treatment intensity based on age.

In conclusion, the analysis shows a clear association between patients’ age and type of surgery, transitioning from more conservative procedures at younger ages to radical interventions in older patients. This information may help to optimize therapeutic strategies and tailor treatment according to age and disease stage.

Statistical analysis shows that patients’ age is the main factor influencing the likelihood of a mastectomy. Adjuvant chemotherapy appears to reduce this likelihood, suggesting a greater likelihood of opting for more conservative interventions. In contrast, neoadjuvant chemotherapy slightly increases the likelihood of a mastectomy, but its impact is not major. Regarding the overall distribution of surgery, no significant differences were identified between patients who received it and those who did not receive chemotherapy.

The analysis did not identify statistically significant differences between age groups and the therapeutic strategies applied.

Adjuvant chemotherapy and adjuvant radiotherapy were administered similarly between age groups, with no major variations.

Neoadjuvant hormone therapy shows a trend towards more frequent use in elderly patients, but this association is statistically weak.

Molecular therapy and CDK4/6 inhibitors were used in a limited number of cases, and their distribution was not significantly influenced by age.

## Figures and Tables

**Table 1:** Patient Demographics and Tumor Histopathology by Age Group.

Variable	Values	Age Group (Years)			
		Under 50	51–60	61–70	Over 71
Patients	N	25	18	28	8
	%	31.6	22.8	35.4	10.1
Histological type	ductal in situ	0 (0.00%)	0 (0.00%)	1 (1.30%)	1 (1.30%)
	invasive ductal	20 (25.30%)	17 (21.50%)	20 (25.30%)	5 (6.30%)
	invasive lobular	5 (6.30%)	1 (1.30%)	6 (7.60%)	2 (2.60%)
	lobular and ductal invasive	0 (0.00%)	0 (0.00%)	1 (1.30%)	0 (0.00%)

**Table 2:** Age distribution of palpable tumor cases.

Variable	Value	Age (Years)			
		Under 50	51–60	61–70	Over 71
Palpable tumor	NO	9 (11.39%)	5 (6.33%)	8 (10.13%)	2 (2.53%)
	YES	16 (20.25%)	13 (16.46%)	20 (25.32%)	6 (7.59%)
Pain	absent	12 (15.20%)	10 (12.70%)	19 (24.10%)	3 (3.80%)
	present	13 (16.50%)	8 (10.10%)	9 (11.40%)	5 (6.30%)

**Table 3:** Age distribution of cases presenting with pain and palpable tumors.

Pain	Palpable Tumor	Age (Years)			
		Under 50	51–60	61–70	Over 71
Absent	No	8 (18.20%)	4 (9.10%)	7 (15.90%)	2 (4.50%)
	Yes	4 (9.10%)	6 (13.60%)	12 (27.30%)	1 (2.30%)
Present	No	1 (2.90%)	1 (2.90%)	1 (2.90%)	0 (0.00%)
	Yes	12 (34.30%)	7 (20.00%)	8 (22.90%)	5 (14.30%)

**Table 4:** Distribution of surgical interventions.

Surgeries	Cases
	N (%)
Lymphadenectomy	68 (86.08%)
Mastectomy	60 (75.95%)
Lumpectomy	19 (24.05%)
Sentinel	12 (15.19%)

**Table 5:** Distribution of therapeutic strategy versus age groups.

Therapy		Age Class (Years)				*p*
		Under 50	51–60	61–70	Over 71	
Adjuvant chemotherapy	NO	20 (25.3%)	13 (16.5%)	20 (25.3%)	5 (6.3%)	0.774
	YES	5 (6.3%)	5 (6.3%)	8 (10.1%)	3 (3.8%)	
Adjuvant radiotherapy	NO	9 (11.4%)	6 (7.6%)	15 (19.0%)	4 (5.1%)	0.454
	YES	16 (20.3%)	12 (15.2%)	13 (16.5%)	4 (5.1%)	
Neoadjuvant hormone therapy	NO	25 (31.6%)	16 (20.3%)	25 (31.6%)	6 (7.6%)	0.155
	YES	0 (0.0%)	2 (2.5%)	3 (3.8%)	2 (2.5%)	
Adjuvant hormone therapy	NO	3 (3.8%)	2 (2.5%)	5 (6.3%)	2 (2.5%)	0.169
	YES	22 (27.8%)	16 (20.3%)	23 (29.1%)	6 (7.6%)	
Inhibitors CDK4/6	No	20 (25.3%)	14 (17.7%)	23 (29.1%)	7 (8.9%)	0.944
	YES	5 (6.3%)	4 (5.1%)	5 (6.3%)	1 (1.3%)	
Molecular targeted therapy	NO	22 (27.8%)	15 (19.0%)	23 (29.1%)	7 (8.9%)	0.935
	YES	3 (3.8%)	3 (3.8%)	5 (6.3%)	1 (1.3%)	

## Data Availability

The authors declare that the data of this research are available from the corresponding authors upon reasonable request.

## Abbreviations

The following abbreviations are used in this manuscript:

IHC	immunohistochemical
DCIS	ductal carcinoma in situ
BCS	breast-conserving surgery
WHO	World Health Organization
CT	Computed Tomography
MRI	Magnetic Resonance Imaging
SPSS	Statistical Package for Social Sciences
SD	standard deviation
BC	breast cancer
NAC	neoadjuvant chemotherapy
SLNB	sentinel lymph node biopsy
ALND	axillary lymph node dissection
